# Stemness of Human Pluripotent Cells: Hypoxia-Like Response Induced by Low Nitric Oxide

**DOI:** 10.3390/antiox10091408

**Published:** 2021-09-02

**Authors:** Estefanía Caballano-Infantes, Irene Díaz, Ana Belén Hitos, Gladys Margot Cahuana, Antonio Martínez-Ruiz, Bárbara Soria-Juan, Rosario Rodríguez-Griñolo, Abdelkrim Hmadcha, Franz Martín, Bernat Soria, Juan R. Tejedo, Francisco Javier Bedoya

**Affiliations:** 1 Department of Regeneration and Cell Therapy, Andalusian Center for Molecular Biology and Regenerative Medicine (CABIMER), University of Pablo de Olavide-University of Seville-CSIC, 41013 Seville, Spain; irene.diaz@cabimer.es (I.D.); ana.hitos@cabimer.es (A.B.H.); khmadcha@upo.es (A.H.); fmarber@upo.es (F.M.); jrtejhua@upo.es (J.R.T.); 2 Department of Molecular Biology and Biochemical Engineering, Universidad Pablo de Olavide, 41013 Seville, Spain; gmcahmac@upo.es; 3 Biomedical Research Network for Diabetes and Related Metabolic Diseases-CIBERDEM, Instituto de Salud Carlos III, 08036 Madrid, Spain; bsoresc@acu.upo.es; 4 Unidad de Investigación, Hospital Universitario Santa Cristina, Instituto de Investigación Sanitaria Princesa (IIS-IP), E-28009 Madrid, Spain; amartinezruiz@salud.madrid.org; 5 Fundación Jiménez Díaz Health Research Institute, 28040 Madrid, Spain; barbara.soria.juan@hotmail.com; 6 Departamento de Economía, Métodos Cuantitativo e Historia Económica, Universidad Pablo de Olavide, 41013 Seville, Spain; mrrodgri@upo.es; 7 ISABIAL and Institute of Bioengineering, University Miguel Hernández de Elche, 03010 Alicante, Spain

**Keywords:** pluripotency, normoxia, nitric oxide, hypoxia, metabolism

## Abstract

The optimization of conditions to promote the stemness of pluripotent cells in vitro is instrumental for their use in advanced therapies. We show here that exposure of human iPSCs and human ESCs to low concentrations of the chemical NO donor DETA/NO leads to stabilization of hypoxia-inducible factors (HIF-1α and HIF-2α) under normoxia, with this effect being dependent on diminished Pro 402 hydroxylation and decreased degradation by the proteasome. Moreover, the master genes of pluripotency, NANOG and OCT-4, were upregulated. NO also induces a shift in the metabolic profile of PSCs, with an increased expression of hypoxia response genes in glycolysis. Furthermore, a reduction in the mitochondrial membrane potential with lower oxygen consumption and increased expression of mitochondrial fusion regulators, such as DRP1, was observed. The results reported here indicate that NO mimics hypoxia response in human PSCs and enhances their stemness properties when cultured under normoxic conditions.

## 1. Introduction

Pluripotent stem cells (PSCs) display the capacity to proliferate while maintaining the ability to generate different cell types during organ development and tissue regeneration [1]. The molecular mechanisms underlying these features are being extensively studied given the potential applications of PSCs in cell therapy. Additionally, these cells display a distinct metabolic signature with poorly active mitochondria and predominant anaerobic glycolysis [2,3,4,5]. Upon differentiation, PSCs undergo changes that favor aerobic metabolism [6]. The relevance of this metabolic shift is underlined by the changes mitochondria experience during cell reprogramming in a process called rejuvenation [2]. Mitochondrial fusion is regulated by GTPase proteins mitofusin 1 (MFN1) and 2 (MFN2) and OPA1 [7,8]. Mitochondrial fission is regulated by DRP1, which is a cytosolic protein that interacts with proteins such as FIS1, mitochondrial fission factor (MFF), and mitochondrial proteins MID49 and MID51 [8,9]. Regarding the relationship between mitochondrial dynamics and the regulation of pluripotency, it has been reported that REX1, a transcription factor associated with pluripotency [10], positively regulates DRP1 activation in a CDK1/cyclin B-dependent process [11]. Moreover, it has been described that increased MNF2 and OPA1 expression and inhibition of fission are required for differentiation into cardiomyocytes [12,13]. This evidence supports a role for mitochondrial dynamics during cell reprogramming and differentiation. A role for oxygen tension in the regulation of stem cell biology has been put forward since low oxygen tension (hypoxia) prevents differentiation of human ESCs and other PSCs [14]. Indeed, a hypoxic environment is a salient feature of the stem cell niche [15]. In this scenario, PSCs rely on glycolysis to produce ATP [2,3,4]. Hypoxia elicits a wider metabolic response orchestrated by hypoxia-inducible factors (HIFs 1–3). Functionally active HIFs are composed of two subunits, the constitutive beta subunit (HIF-β) and the regulatory alpha subunit (HIF-α) that accumulates swiftly in hypoxia [16,17]. HIF-1α levels are mainly regulated by post-translational hydroxylation of proline residues (Pro-564 and Pro-402) in reactions catalyzed by prolyl hydroxylases (PHD1, PHD2, and PHD3). Hydroxylated HIF-α is targeted for ubiquitination by E3 ubiquitin protein ligase containing Von Hippel–Lindau protein (VHL). Hypoxia also enhances the generation of inducible pluripotent stem cells (iPSCs) [18,19], and it has been reported that reprogramming cells to pluripotency requires HIF-1α and HIF-2α at an early state to induce the metabolic switch from an oxidative to a highly glycolytic state, as well as for the acquisition of an undifferentiated state [19,20]. The evidence thus reviewed indicates that changes in metabolism are relevant for transition from the pluripotent to the differentiated state. In this regard, the gas messenger nitric oxide (NO) has been reported to regulate mitochondrial function by binding to cytochrome c oxidase [21,22]. Thus, at low oxygen concentrations, low levels of NO allow cells to become dependent on glycolysis [23,24,25]. In addition, we previously reported that low concentrations of the chemical NO donor DETA-NO induce the expression of pluripotency genes Oct4, Nanog and Sox2 and prevent the expression of differentiation genes Gata-4, Gata-6, Brachyury, Fgf5 and Fgf8 in both mouse and human embryonic SCs [26].

It is appealing to hypothesize a role for NO in the regulation of metabolism in PSCs. The results shown here indicate that production of low amounts of NO generates a metabolic state in human PSCs similar to hypoxia that could be instrumental for the maintenance of the pluripotent phenotype.

## 2. Materials and Methods

### 2.1. Cell Culture and Treatment

**Figure 1:**
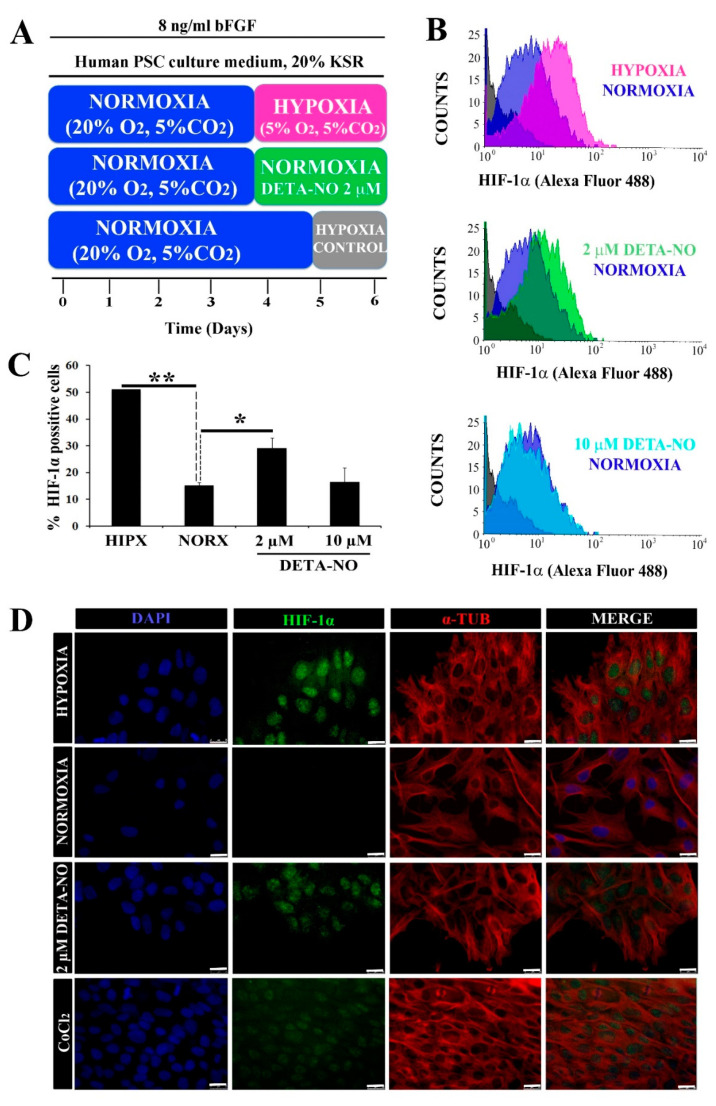
Low doses of DETA-NO induce the accumulation of HIF-1α protein in human iPSCs cultured under normoxia. (**A**) Cells from the MSUH-001 line were cultured in fibroblast-conditioned human PSC culture medium and supplemented with 20% KSR and bFGF (8 ng/mL) for 6 days according to the culture protocols shown. (**B**) Analysis of HIF-1α by flow cytometry. Cells were treated for 48 h with low doses of DETA-NO (2 μM and 10 μM) in normoxia and 48 h in 5% hypoxia O_2_. The intensity of the HIF-1α fluorescent signal is plotted against the number of cells counted. Each plot is representative of 3 independent experiments. The isotype control and secondary antibody control that were depicted in gray were used as negative controls. The number of cells tested is at least 3000–5000 cells in each experimental condition. (**C**) Percentage of cells positive for HIF-1α ± SEM of 3 independent experiments. (**D**) Immunocytochemical localization of HIF-1α. Cells were labeled with DAPI (blue), anti-HIF-1α (green), and anti-α-Tubulin (red). Scale bar: 25 μm. The images shown are representative of 3 independent experiments and have been acquired with the Leica DM6000B fluorescence microscope (AF6000). (*) *p* < 0.05; (**) *p* < 0.0001. HIPX (HYPOXIA); NORX (NORMOXIA); KSR (KnockOut Serum Replacement).

Human induced PS cell line MSUH-001 and human ESC line HS181 were used. MSUH-001 cells were derived from the line of fetal fibroblasts IMR90 (ATCC: CCL-186; Manassas, VA 20110-2209, USA) and were provided by the Cellular Reprogramming Laboratory (Michigan State University, East Lansing, MI, USA) and assigned to the Andalusian Stem Cell Bank (Granada, Spain). The human ESC line HS181 was generated by O. Hovatta and provided by the Karolinska Institute (Solna, Sweden). Cells were formally acquired by the Andalusian Center for Molecular Biology and Regenerative Medicine (Sevilla, Spain). Both human cell lines were cultured in a conditioned medium containing Knockout D-MEM (Gibco, Waltham, MA, USA), replacement serum (Gibco), 5000 U/mL penicillin and 5000 µg/mL streptomycin (Gibco), 2 mM L-glutamine (Gibco), 0.1 mM nonessential amino acids (Gibco) and 0.5 mM β-mercaptoethanol (Gibco). Conditioned medium was obtained by culturing human fibroblasts (ATCC: CRL2429) in Iscove’s Modified Dulbecco’s Medium (IMDM) (Gibco) supplemented with 10% inactive FBS as well as 25 U/mL penicillin and 25 μg/mL streptomycin. After reaching 80% confluence, cells were inactivated by exposure to 10 ng/mL mitomycin (Sigma-Aldrich, St. Louis, MO, USA) for 3 h. Following medium removal and two washes with Dulbecco’s phosphate buffered saline (DPBS), fibroblasts were further cultured for 24 h. The medium was then removed and stored at −20 °C. Human PSCs were grown on plates coated with Matrigel (BD Biosciences, Franklin Lakes, NJ, USA) for 6 days in a conditioned medium supplemented with basic fibroblast growth factor (bFGF) (Invitrogen, Waltham, MA, USA) at a final concentration of 8 ng/mL under normoxic conditions (20% O_2_). The medium was renewed daily to avoid spontaneous differentiation. Subsequently, cells were dispersed with acutase (eBioscience, San Diego, CA, USA) at 37 °C and collected by centrifugation at 800 rpm. In the protocol for hypoxia experiments (Figure 1A), cells cultured for 4 days under normoxia were subsequently exposed to 5% O_2_ for 48 h. When appropriate, cells were exposed to the chemical NO donor diethylenetriamine nitric oxide adduct (DETA/NO) (Sigma-Aldrich) for 48 h at 2 and 10 µM. The DETA/NO concentrations tested were selected according to previous dose-response studies carried out in our laboratory. In control experiments, chemical hypoxia response was induced by exposing cells cultured under normoxia to either 150 μM CoCl_2_ or 1 mM DMOG (ENZO Life Sciences, New York, NY, USA) for 4 h.

### 2.2. RNA Isolation

RNA was isolated with Easy Blue reagent (Intron Biotechnology) and chloroform/isopropanol precipitation and washed with 75% ethanol. RNA was then stored at –80 °C. RNA was quantified with a NanoDrop ND-1000 spectrophotometer.

### 2.3. Reverse Transcription and Real-Time qPCR

cDNA synthesis was carried out with 1μg of total RNA with retrotranscriptase enzyme (Quanta Biosciences) according to the manufacturer’s instructions. The cDNA obtained was diluted with molecular grade water and then used as a template for quantitative PCR (primer melting temperature “Tm” for all genes was designed at 60 °C) using SYBR Green 2X (iTaq Universal SYBR Green One-Step Kit, Bio-Rad, CA, USA). Detection was performed with the ABI Prism7500 machine (Applied Biosystems). All samples were normalized with a housekeeping gene (β-actin), used as loading control and analyzed with the ∆∆CT algorithm. The results show an average of *n* ≥ 3 independent experiments. Data are presented as mean ± SEM. The experiments with *n* = 2 are represented as median with individual data points. The *y*-axis corresponds to relative expression level of genes assessed in this study (fold change). Here, we focused on the changes in mRNA expression between low NO treatment and normoxia conditions without NO. Because of this, we have relativized the results to normoxia control. Primers are listed in Supplementary Table S1.

### 2.4. Western Blotting

Cells were removed from plates by scraping in cold phosphate buffered saline (PBS, Gibco). The process was carried out on ice because HIF-1α and HIF-2 α proteins degrade rapidly in normoxia. Cells were collected by centrifugation at 800 rpm for 5 min and pellets were washed with PBS and suspended in RIPA buffer (Sigma) supplemented with Protease and Phosphatase Inhibitor Cocktail (Sigma). Lysis consisted of incubation for 45 min on ice and sonication by 4 pulses (10 s each) at 10% amplitude in a sonifier (Branson Ultrasonics Corporation, Brookfield, CT, USA). Cell sonicates were centrifuged and protein supernatants were quantified by Bradford assay. Samples were denaturalized with Laemmli buffer containing 2.5% β-mercaptoethanol for 10 min at 96 °C. SDS-PAGE was carried out for protein separation and subsequent transfer to PVDF membranes (Amersham, Hybond). Membranes were then blocked with either 5% defatted dried milk in PBS (Gibco) or 3% of BSA in Tris buffered saline (TBS)-Tween.

Antibodies were anti-HIF-1α 1:250 (Santa Cruz, 10790); anti-HIF-1α 1:500 (Abcam,); anti-HIF-2α 1:500 (Abcam); anti-NANOG 1:500 (Cell Signaling); anti-OCT4 1:500 (Cell Signaling) and anti-β-actin 1:10,000 (Sigma). Secondary antibodies were anti-rabbit IgG 1:20,000 (Sigma) and anti-mouse IgG 1:40,000 (Jackson ImmunoResearch). Protein blots were visualized by chemiluminiscence with Western Bright ECL HRP substrate (Advansta) according to the manufacturer´s instructions. Western blots were quantified by densitometry using Photoshop CS4. In several experiments, blots were quantified by near-infrared fluorescence imaging (Odissey CLx. Li-COR Biosciences).

### 2.5. Flow Cytometry

To identify HIF-1α- and HIF-2α-expressing cells by cytometry, cells were detached with a scraper and collected with cold PBS. Cells were then fixed with 4% paraformaldehyde (PFA) at pH 7.4 for 15 min at 4 °C and 5 min at room temperature (RT). Cells were permeabilized with 0.3% Triton X-100 for 15 min on ice and blocked with 5% FBS at 4 °C for 30 min. Cells were incubated at 4 °C for 1h with anti-HIF-1α 1:100 (Abcam), anti-HIF-2α 1:100 (Abcam), anti-HIF-1α (OH P402) 1:100 (Abcam), and anti-NANOG 1:500 (Cell Signaling). Cells were then washed 3 times in PBS and incubated with anti-mouse Alexa fluor 488 1:500 (Invitrogen,), anti-rabbit Alexa fluor 633 1:500 (Invitrogen,), and anti-rabbit IgG-PE 1:500 (Abcam) for 30 min at RT. Cells were then washed 3 times at 4 °C with PBS and resuspended in 500 mL of PBS. Fluorescence analysis was carried out by flow cytometry (FACSCalibur; BD Biosciences). In this assay, we use as an isotype control the following antibodies: anti-rabbit IgG phycoerythrin (PE) goat isotype (Abcam); rabbit control IgG (Abcam), and mouse IgG1 isotype control mAb (Cell Signaling). The percentage of positive HIF-1α and Hif-2α cells was calculated by FACSCalibur software after we selected a correct cell gate. The program analyzes the percentage of positive HIF-1α and Hif-2α cells.

### 2.6. Immunocytochemistry

Cells were seeded on coverslips as follows. Glass coverslips 24-mm in diameter were washed with 70% ethanol and exposed to UV radiation for 1h. Coverslips were air-dried and placed in 6-well tissue culture plates (Nunc). The plates were coated with Matrigel for 1 h at RT. Next, coverslips were washed with Knockout Dulbecco’s modified Eagle’s medium and seeded with cells at a density of 5 × 10^3^ cells/well. Then, cells were washed with ice-cold PBS and fixed with 4% paraformaldehyde for 20 min at RT, followed by permeabilization with PBS containing 0.01% saponin at RT for 30 min. We used blocking solution for 30 min for NANOG (100 mM glycine PBS) and 60 min for HIF-1α and HIF-2α (0.3 mM glycine in PBS, pH 7.2), then incubated these samples overnight at 4 °C with rabbit anti-NANOG (Bethyl Laboratories), rabbit anti-HIF-1α, and rabbit anti-HIF-2α. For detection of primary antibodies, anti-mouse Alexa Fluor 488 (Invitrogen), anti-rabbit Alexa Fluor 594 (Invitrogen), and anti-goat Alexa Fluor 488 (Invitrogen) were used. Cells were stained with 300 nM DAPI (Roche). Coverslips were mounted on microscope slides with VECTASHIELD mounting medium (Vector Laboratories) and visualized with Zeiss Apotome.

### 2.7. Cell Proliferation

5-Bromo-2-deoxyuridine (BrdU) incorporation into DNA was studied with the BrdU “Cell Proliferation Elisa BrdU colorimetric” (Roche, Basel, Switzerland). 300–500 cells were seeded by Cell Sorter (BD FACSAria) into 96-well plates on a Matrigel layer, preparing 3–6 replicates per experimental condition. Briefly, cells were exposed to BrdU (1:100) for 16 h before the end of exposure to hypoxia or DETA/NO. Subsequently, cells were fixed and exposed to the anti-BrdU antibody (1:100) for 2 h at RT. To allow the antibody to bind to the BrdU incorporated into the DNA, cells must be pre-fixed, permeabilized and DNA-denatured through Kit fixing solution (Fixdenat, Roche) for 30 min at RT. To remove the excess antibody, 3 washes with 200 μL of wash solution included in the kit were performed. Cells were then incubated with the substrate solution for 5–30 min at RT to finally stop the reaction with H_2_SO_4_. Finally, absorbance at 450 nm was measured which is proportional to the degree of cell proliferation.

### 2.8. Mitochondrial Membrane Potential

To evaluate mitochondrial membrane polarization in the human PSC line, 104 cells were seeded on Matrigel-coated coverslips at a final dilution of 1:60. At the end of the treatment, the cells were incubated in a culture medium with the mitochondrial marker MitoTracker Red 1 μM (ThermoFisher Scientific) for 30 min at 37 °C. After incubation cells were washed 2 times for 30 min with a culture medium and then fixed with 4% PFA for 10–20 min. After fixation, cells were washed with PBS to remove excess PFA, and nuclei were visualized with DAPI (1 μg/mL) (Roche). Fluorescence analysis was performed by fluorescence microscopy and flourescence was estimated using the DeltaVision software (GE Healthcare UK Limited Amersham Place, Little Chalfont Buckinghamshire, HP7 9NA UK).

Alternatively, quantification of Δψm was performed by flow cytometry using the mitochondrial fluorescent marker MitoTracker Deep Red FM (Thermo Fisher Scientific, Waltham, MA, USA). Cells were grown at 50–75% confluence in a 75 cm^2^ flask. Cells were exposed to the dye for 30 min at 37 °C. Following 2–3 washes with culture medium, cells were fixed with PFA 4%. After fixing, cells were washed with PBS, collected by scraping, and analyzed using a FACStar flow cytometer (BD FACSCalibur).

The mitochondrial marker JC-1 was also used (Sigma-Aldrich). This compound appears as monomers when Δψm is low, emitting green fluorescence, or in aggregate to form dimers when Δψm is high, emitting red fluorescence. To measure the Δψm, 5–10 × 10^3^ cells were seeded in 6-well plates. At the end of the treatment, the JC-1 marker was incubated at a concentration of 2 μM for 30 min in living cells at 37 °C in a serum-free medium. FCCP was used as an uncoupling agent to induce low Δψm at 5 µM (FCCP was incubated at the same time as JC-1). Cells were washed with PBS, collected by scraping, pelleted by centrifugation, and re-suspended in 500 µL PBS. Cells were analyzed by flow cytometry by measuring both green and red fluorescence. Relative degrees of mitochondrial polarization were quantified using a FACStar flow cytometer (BD FACSCalibur) by measuring the ratio of red-shifted JC-1 aggregates, which are favored under conditions of high membrane potential, and green-shifted monomers, which tend to predominate under conditions of low membrane potential.

### 2.9. OCR and ECAR Measurements

To study the effect of NO on mitochondrial energetics, O_2_ consumption rate (OCR) and extracellular acidification rate (ECAR) were studied with the Seahorse XF24 analyzer (Seahorse Bioscience, Massachusetts, USA). HS181 cells were seeded onto Matrigel-coated Seahorse plates. After 48 h, cells were washed 3 times with 0.9 Na Cl %. Subsequently, 675 µL of Seahorse medium (unbuffered DMEM, Sigma) was added. Plates were pretreated for 1 h at 37 °C in CO_2_-free conditions to reach temperature and pH equilibrium. Then, port 1 was loaded with basal medium, port 2 was loaded with oligomycin at a final concentration of 6 µM, port 3 was loaded with FCCP (0.3 µM) and port 4 was loaded with rotenone and antimycin (0.1 μM). The mitochondrial stress protocol started by measuring baseline oxygen consumption rate (OCR) followed by measurement of OCR changes in response to injection of oligomycin, FCCP, and finally antimycin and rotenone. Oxygen consumption and ECAR were measured with the Seahorse. Basal respiration was defined as the average values measured from time point 1 to 5 (0–45 min). ATP production, proton leak, and non-mitochondrial respiration were calculated according to Seahorse XF cell mito stress parameter equations. Data were normalized to the amount of protein present in each well, which was quantified by BCA (Pierce BCA Protein Assay Kit (Thermo Fisher Scientific)).

### 2.10. Proteasome Assay

The Proteasome Activity Assay Kit (CHEMICON) was used for the detection of proteasome activity in cell lysates. The assay is based on the detection of AMC (7-Amino-4-methyl coumarin) after its separation from the LLVY substrate due to proteasome activity. The fluorescence emitted by AMC is quantified by using 380/460 nm filters in a fluorometer. As a control of the technique, the kit provides a proteasome inhibitor, lactacystin. Additionally, we have used the MG132 protease inhibitor (Sigma). Briefly, a standard curve with AMC was prepared, and emitted fluorescence was measured in a Varioskan Flash microplate fluorometer (Thermo Fisher Scientific). The kit also has positive control of the 20S proteasome, which was prepared according to the manufacturer’s instructions. A standard curve was then prepared and 10 μL of the 1:20 diluted substrate was added. Incubation was carried out at 37 °C for 2 h. Concerning sample preparation, once collected, the cells were resuspended in 50 mM HEPES pH 7.5, 5 mM EDTA, 150 mM NaCl and 1% Triton x-100 lysed for 30 min on ice with shaking every 10 min. Then, they were centrifuged at 14,000 rpm for 15 min and supernatants containing 15–30 μg protein were used for analysis. After incubation for 2 h at 37 °C, fluorescence was measured in a microplate fluorometer (Varioskan Flash, Thermo Fisher Scientific). Results were plotted as units of fluorescence emitted in each experimental condition and relativized to the condition of normoxia.

### 2.11. ROS Measurements

O_2_^−^ production by mitochondria was followed by oxidation of fluorogenic dye MitoSox Red (Thermo Fisher Scientific). 5000–10,000 cells were seeded in 6-well plates. The medium was removed at the end of the treatment period and washed cells were incubated with serum-free medium containing 2 μM MitoSox Red at 37 °C for 30 min. After the incubation, the medium was removed, and cells were washed with PBS. Cells were then detached with acutase. Centrifugation at 800–1000 rpm for 5 min ensued, and cell pellets were suspended in 500 μL of PBS. Cells that were not exposed to MitoSox were used as negative controls. As a positive control, cells exposed to 200 μM H_2_O_2_ for 1 h were used. The analysis was carried out on a FACsCalibur cytometer fitted with BD Cell Quest Pro Software (San Jose, CA, USA).

### 2.12. Statistical Analysis

Data are presented as mean ± standard error of the mean (SEM). To determine statistical significance, non-parametric tests were performed, and *p* < 0.05 was considered significant. Wilcoxon and Kruskal–Wallis tests were carried out. The data were statistically analyzed using JMP Software (version PRO 14, SAS Campus Drive Cary, NC, USA).

## 3. Results

### 3.1. Exposure to NO Stabilizes HIF Proteins and Enhances Pluripotency

To study the role of NO on HIF-1α protein stabilization in normoxia, the expression of this protein was analyzed by flow cytometry. When human iPSCs (MSUH-001) were exposed to the protocol depicted in Figure 1A, a significant increase in HIF-1α-positive cells in the hypoxia condition (5% O_2_) was apparent when compared with cells cultured under normoxia (20% O_2_). Upon exposure to 2 µM DETA-NO for an additional period of culture under normoxia, the percentage of HIF-1α-positive human iPSC cells rose significantly in comparison with normoxia alone (Figure 1B,C). NO concentrations and the incubation period were previously optimized from a dose and time dependency study. DMOG and CoCl_2_ were used as positive controls for HIF-1α accumulation. Here we found, as can be seen in Figure 1B,C, that only the lower dose (2 µM) induced stables levels of HIF-1α in normoxia, mimicking the hypoxia response. Thus, 2 μM DETA-NO was selected as the reference dose because of the positive effect on HIF-1α accumulation. Furthermore, exposure to the NO donor promoted nuclear accumulation of HIF-1α in normoxia (Figure 1D).

Low NO also induced accumulation of HIF-1α protein in human ESC when studied by Western blotting and fluorescence microscopy (Supplementary Figure S1A–C).

**Figure 2:**
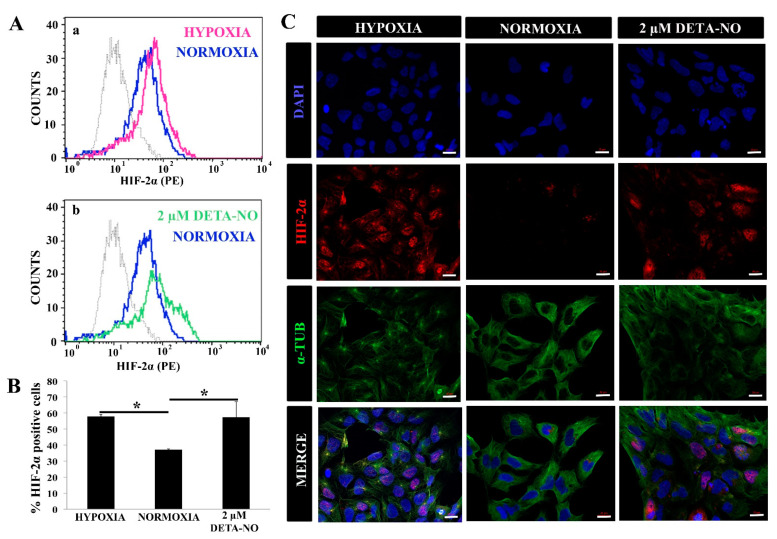
Exposure to 2 μM DETA-NO induces HIF-2α accumulation under normoxia. (**A**) Quantification by flow cytometry. Fluorescence intensity for HIF-2α is plotted against the number of cells. Each plot is representative of three independent experiments. Isotype (IgG) and secondary antibody (PE) shown in gray were used as negative controls. In the study, 5000 cells were analyzed in each experimental condition. (a) Representative cytometry plot of cells cultured under hypoxia condition versus normoxia. (b) Representative cytometry plot of cells treated with low NO in normoxia condition versus normoxia without NO. (**B**) Percentage of HIF-2α ± SEM-positive cells from three independent experiments is shown. (*) *p* < 0.05. (**C**) Exposure to low doses of DETA-NO induces nuclear accumulation of HIF-2α in normoxia. In the figure, the human induced PS cell line from the MSUH-001 line was observed after 6 days of culture and treatment of 48 h of hypoxia (5% of O_2_) and 48 h of exposure to DETA-NO 2 μM under normoxia conditions. Cells were labeled with DAPI (blue), anti-HIF-2α (red), and anti-α-Tubulin (green). Scale bar: 25 μm. The images shown are representative of two independent experiments and were acquired with the Zeiss ApoTome fluorescence microscope.

The effect of NO on HIF-2α stabilization was also studied. Flow cytometry studies revealed that treatment for 48 h with 2 μM DETA-NO significantly increased the number of HIF-2α positive human iPSC cells (Figure 2A,B). Next, we proceeded to study the cellular localization of HIF-2α protein under different experimental conditions. Both hypoxic cells and DETA-NO- treated cells in normoxia increase nuclear localization of the HIF-2α protein (Figure 2C).

Similar results were obtained in the HS181 hESC line when studied by Western blotting, flow cytometry and fluorescence microscopy (Supplementary Figure S2A–E).

**Figure 3:**
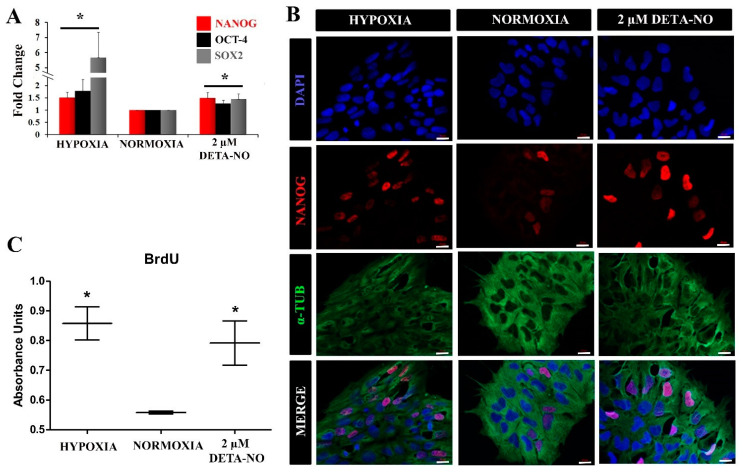
Exposure to low doses of DETA-NO increases the expression of pluripotency markers and improves cell proliferation. (**A**) Analysis of the expression of NANOG and OCT-4 by qPCR. The plot shows the mean of the relative expression of 5 independent experiments ± SEM. The relative expression of these genes has been obtained using the algorithm ∆∆ Ct. β-actin expression is used as a housekeeping gene and data were relativized to the expression in normoxia. (*) *p* < 0.05. (**B**) Exposure to low doses of DETA-NO increases the expression of NANOG with nuclear localization. After 6 days of culture under normoxia, MSUH-001 cells were exposed to hypoxia (5% of O_2_) for 4 days and to low NO treatment for 48 h. NANOG-positive cells were visualized by fluorescence microscopy. Cells were labeled with DAPI (blue), anti-NANOG (red), and anti-α-Tubulin (green). The images shown are representative of more than 12 planes per experimental condition of 2 independent experiments, and they have been acquired with the Zeiss ApoTome fluorescence microscope. Scale bar: 25 μm. (**C**) Cell proliferation measurement by BrdU incorporation. The graph represents the median with individual data points of 2 independent experiments with at least 4 replicates for each experimental condition. (*) *p* < 0.05.

Next, the impact of NO on human iPSC pluripotency was evaluated. For this, the expression of pluripotency markers in cells cultured under normoxia in the presence of DETA-NO was compared with the pattern in cells cultured under normoxia. The expression of these pluripotency markers was also studied in the hypoxia condition. Quantitative RT-PCR analysis showed that expression of NANOG and OCT-4 increased significantly in cells exposed to 2 µM DETA-NO under normoxia and in cells cultured under hypoxia (5% O_2_) (Figure 3A). The increase in gene expression under DETA-NO was slight, although significant. Up-regulation of NANOG was also apparent by immunocytochemistry (Figure 3B). A similar effect was observed in human ESCs when studied by Western blotting (Supplementary Figure S3A,B). Furthermore, both cells exposed to DETA-NO under normoxia and cells cultured under hypoxia exhibited higher and more significant incorporation of BrdU than cells cultured in normoxia (Figure 3C). These results indicate that the addition of the NO donor to the culture medium improves the proliferation of human iPSCs.

### 3.2. NO Treatment in Normoxia Shifts the Metabolic Profile of Human PSCs towards Glycolysis

**Figure 4:**
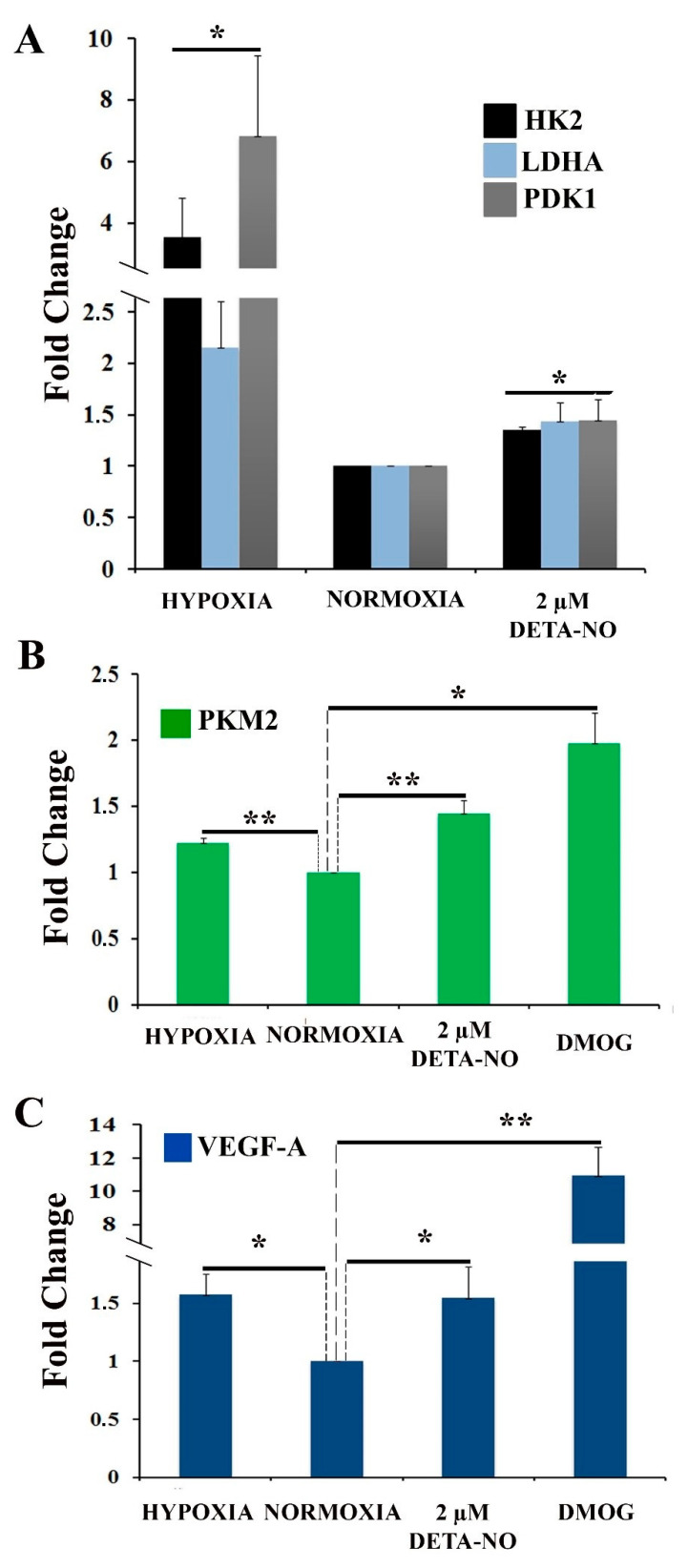
Exposure to 2 μM DETA-NO induces an increase in mRNA level of HIF-1α target genes in normoxia. MSUH-001 cells were cultured in normoxia for 6 days and subsequently exposed for 48 h to either 2 μM DETA-NO under normoxia or hypoxia alone. When appropriate, cells were exposed for 4 h to the hypoxia inducer DMOG (1mM) under normoxia. (**A**) mRNA levels of glycolytic genes HK-2 (hexokinase 2), LDHA (lactate dehydrogenase A), and PDK1 (pyruvate dehydrogenase kinase 1) were measured by qPCR. Plotted data are the mean relative expression ± SEM of 4 independent experiments for HK-2 and LDHA and 5 experiments for PDK1. (**B**) Analysis of the relative expression of PKM2 (pyruvate kinase M1/2) by qPCR. The plot shows the mean of the relative expression ± SEM of 3 independent experiments. (**C**) Analysis of the relative expression at VEGF-A mRNA level by qPCR. The plot shows the mean of the relative expression ± SEM of 3 independent experiments. Expression values were normalized to the expression of the β-actin gene. Values were relativized to the normoxia control. (*) *p* < 0.05; (**) *p* < 0.001.

To assess the effect of NO on cellular metabolic profile, the expression of genes involved in the hypoxia response was analyzed. Hexokinase 2 (HK-2), lactate dehydrogenase A (LDH-A), and pyruvate dehydrogenase kinase 1 (PDK1) mRNA were increased in cells treated with NO and in cells cultured in hypoxia for 48 h compared to normoxia control (Figure 4A). The increase induced by nitric oxide treatment was statistically significant, although much lower than that induced by hypoxia. Next, we studied the effect of NO on the expression of pyruvate kinase muscle isozyme M2 (PKM2) gene. The results show that treatment with DMOG, an inhibitor of prolyl hydroxylase (PHD) activity, increases the level of PKM2 gene expression under normoxia conditions as does treatment with 2 μM DETA-NO and hypoxia (5% O_2_) (Figure 4B). Furthermore, the mRNA expression of VEGF-A, a target gene of HIF-1α, is also increased under the tested experimental conditions. (Figure 4C).

### 3.3. NO Reduces Oxygen Consumption in Human ECSs

**Figure 5:**
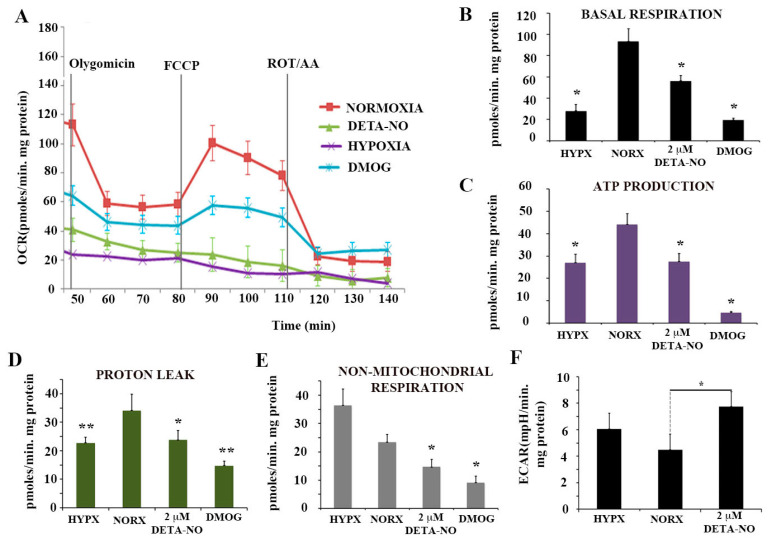
Nitric oxide effects on mitochondrial function. (**A**) Determination of the O2 consumption rate (OCR) in hESCs exposed to hypoxia for 48 h, to 2 μM DETA-NO under normoxia, and to 1 mM DMOG for 4 h in normoxia. Oligomycin (6 μM), FCCP (0.3 μM) and ROT (0.1 μM) + AA (0.1 μM) were added sequentially. (**B**) Basal respiration. (**C**) Production of ATP. (**D**) Proton leakage. (**E**) Non-mitochondrial respiration. The results represent the mean of 2 experiments ± SEM, with at least 5 replicates for each experimental condition. (**F**) Determination of extracellular acidification rate (ECAR) in hESCs. The results represent the mean of 5 independent experiments ± SEM. (*) *p* < 0.05. HIPX (Hypoxia); NORX (Normoxia). (*) *p* < 0.05; (**) *p* < 0.005. HIPX (Hypoxia); NORX (Normoxia); FCCP Carbonylcyanide-4-(trifluoromethoxy)phenylhydrazone); DETANO Diethylenetriamine/nitric oxide adduct; DMOG Dimethyloxaloylglycine; ROT (Rotenone); AA (Antimycin A).

To further characterize the action of NO on PSC metabolism, oxygen consumption rate (OCR) and extracellular acidification rate (ECAR), an index of glycolytic flux, were studied. In this set of experiments, cells were exposed to the ATP synthase inhibitor oligomycin and the uncoupler agent FCCP to measure the maximal capacity of mitochondrial electron chain transport. Hypoxic cells and cells exposed to the hypoxia inducer DMOG had lower OCR than cells cultured under normoxia. Cells exposed to 2 μM DETA-NO in normoxia showed a statistically significant decrease in mitochondrial respiration compared to normoxia controls, as occurs in cells cultured in hypoxia and after treatment with DMOG (Figure 5A,B). Mitochondrial respiration is the result of oxygen consumed for ATP synthesis and proton leak. The respiratory component resulting from ATP synthesis is inhibited by oligomycin. Both hypoxia and DETA-NO significantly decreased the oligomycin-sensitive component in OCR (Figure 5C). Proton leak was also affected (Figure 5D). Finally, non-mitochondrial respiration showed a significant decrease following treatment with 2 μM DETA-NO in normoxia and treatment with 1 mM DMOG (Figure 5E).

Extracellular acidification rate (ECAR) measurements showed a significant increase in cells treated with 2 μM DETA-NO in normoxia compared to normoxia controls (Figure 5F). However, hypoxic cells did not increase ECAR levels because of the reoxygenation process during sample manipulations. The results obtained indicate that DETA-NO decreases oxidative phosphorylation in hESCs as it occurs in hypoxia and after treatment with DMOG. Additionally, low doses of NO increases ECAR in these cells, thus suggesting that a shift towards anaerobiosis takes place.

### 3.4. The Mitochondrial Coupling Is Reduced upon Exposure to Low NO

**Figure 6:**
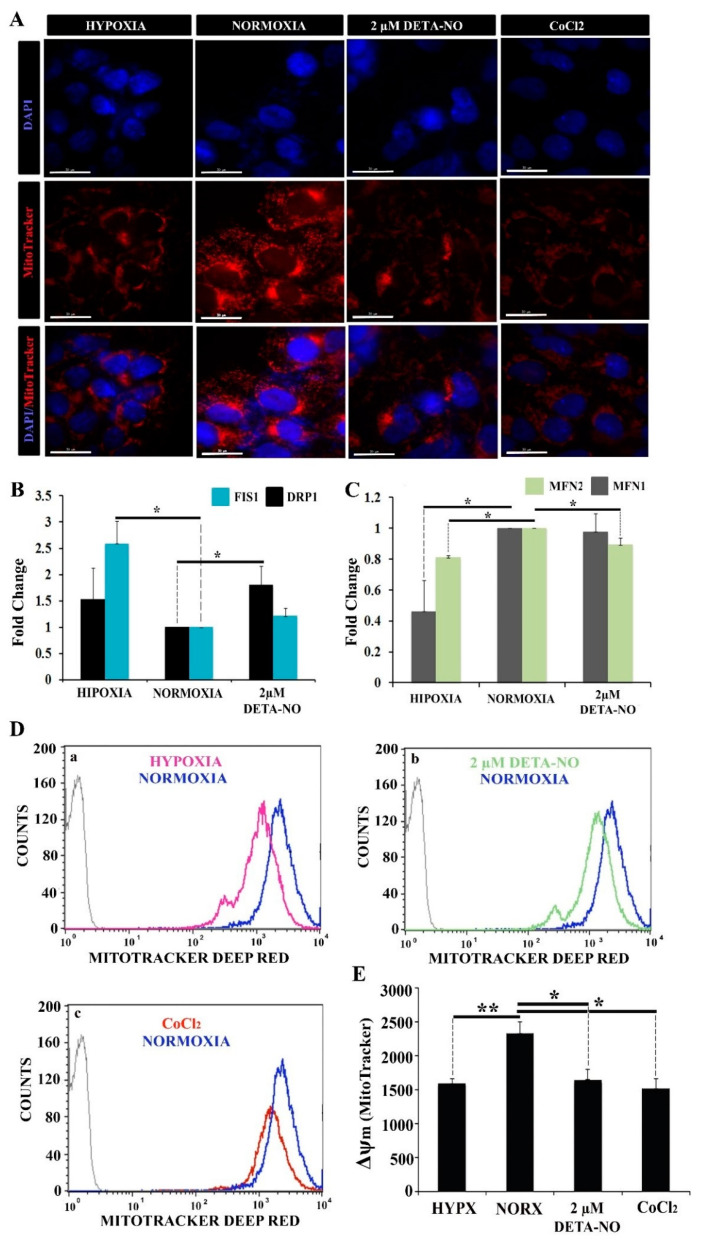
Exposure to 2 μM DETA-NO decreases Δψm in normoxia. (**A**) Treatment with 2 μM DETA-NO in normoxia induces a mitochondrial network distribution similar to hypoxia. Fluorescence microscopy of human iPSCs. MSUH-001 cells were cultured for 6 days in normoxia conditions and subsequently exposed for 48 h to either hypoxia or 2 μM DETA-NO under normoxia. In another set of experiments, cells were exposed to CoCl2 for 4 h in normoxia. Then, cells were incubated for 30 min with MitoTracker Red and fixed in 4% PFA. Detection of nuclear morphology was determined by Hoechst (33342). Scale bar = 30 μm. (**B**) Analysis of mRNA expression of mitochondrial fission markers DRP1 and FIS1. The plot shows the mean of the relative expression of 3 independent experiments ± SEM. (**C**) Analysis of the expression at mRNA level of mitochondrial fusion markers MFN1 and MFN2. The relative expression was calculated with the algorithm ∆∆ Ct. β-actin was measured as a housekeeping gene. (*) *p* < 0.05. (**D**) Analysis of changes in mitochondrial membrane potential (Δψm) by flow cytometry following fluorescent staining with MitoTracker Deep Red. (a) Representative cytometry plot of cells cultured under hypoxia condition versus normoxia. (b) Representative cytometry plot of cells treated with low NO versus cells cultured in normoxia. (c) Representative cytometry plot of cells treated with CoCl_2_ versus normoxia. (**E**) The bar graph represents the mean fluorescence intensity emitted in 6 independent experiments ± SEM. (*) *p* < 0.05: (**) *p* < 0.005. HIPX (HYPOXIA); NORX (NORMOXIA).

Uneven distribution of the mitochondrial network with low perinuclear localization was observed by microscopy in normoxic iPCs. However, in hypoxic cells, the number of mitochondria was reduced, and the localization was predominantly perinuclear. On the other hand, treatment with 2 μM DETA-NO under normoxia conditions induced a hypoxia-like mitochondrial localization showing a lower mitochondrial network dispersion and perinuclear concentration (Figure 6A).

In this experiment, CoCl_2_ was added to mimic hypoxia in stabilizing HIF under normoxia conditions. Expression analysis of mitochondrial fusion markers MFN1 and MFN2 and mitochondrial fission DRP1 and FIS1 was also performed. The results showed a significant increase in DRP1 mRNA in cells treated with 2 μM DETA-NO under normoxia compared to the normoxic control. (Figure 6B). On the other hand, FIS1 mRNA expression increased significantly in cells cultured in hypoxia without significant changes after treatment with 2 μM DETA-NO in normoxia (Figure 6B). Regarding the expression of mitochondrial fusion genes, mRNA levels of MFN1 and MFN2 decreased in hypoxia compared to normoxia control. Moreover, MFN1 levels after treatment with NO remained stable when compared to the expression of this gene in cells cultured in normoxia in the absence of NO. However, MFN2 mRNA expression level decreased in cells treated with 2 μM DETA-NO in normoxia compared to normoxia control in the absence of NO (Figure 6C). The analysis of mitochondrial function was further implemented by measuring changes in membrane potential (Δψm) with MitoTracker Deep Red. The results show that in cells exposed to 2 μM DETA-NO in normoxia, Δψm significantly decreased compared to the normoxic control (Figure 6D,E). A similar pattern is observed in hypoxic hiPSCS and after CoCl2 treatment (Figure 6D,E). Fluorescent staining of mitochondria with the mitochondrial marker JC-1 generated similar results (Supplementary Figure S4A,B).

The collected data indicate that the mitochondrial potential membrane is affected by exposure of cells to DETA-NO.

### 3.5. HIF Hydroxylation Is Critically Affected by NO

**Figure 7:**
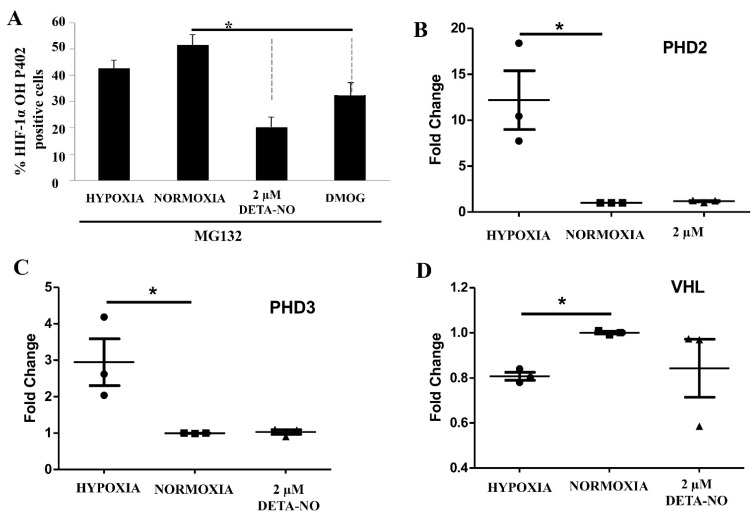
Effect of treatment with 2 μM DETA-NO on HIF stability regulators. (**A**) Quantification by flow cytometry of HIF-1α hydroxylation. MSUH001 cells were cultured for 6 days in a conditioned medium in the presence of bFGF and subsequently exposed to 5% of O_2_ for 48 h. In another set of experiments, cells were cultured under normoxia (20% O_2_) and 2 μM DETA-NO for 48h. Exposure to DMOG (1 mM) for 4 h under normoxia was studied as a positive control. To halt the degradation of hydroxylated HIF-1α, the proteasome inhibitor MG132 (10 μM) was added 4 h before the end of the experiment. The bar graph shows the percentage of HIF-1α OH P402 ± SEM positive cells from 3 independent experiments. (**B**,**C**) Analysis of mRNA expression of prolyl hydroxylases 2 and 3 (PHD2, PHD3). The graph shows the mean of the relative expression of 4 independent experiments ± SEM. (**D**) mRNA expression levels of the Von Hippel–Lindau (VHL) gene. The plot shows the mean of the relative expression of 3 independent experiments ± SEM. The relative expression of genes was calculated with the algorithm ∆∆ Ct. Values were relativized to the expression of the β-actin gene, and values were relativized to normoxia condition. (*) *p* < 0.05; HIF-1α OH P402 (HIF-1α hydroxylated at the proline residue P402).

The cellular content of the HIF-1α protein is mainly controlled by hydroxylation and attendant degradation. Levels of Pro 402 HIF-1α hydroxylation were assessed by cell cytometry upon exposure to NO. To prevent degradation of hydroxylated HIF, the proteasome inhibitor MG132 (10 μM for 4 h) was added. The results show that exposure of normoxic cells to 2 μM DETA-NO led to a significant reduction in hydroxylated HIF-1α when compared with the normoxia alone condition. Here we found that hydroxylated HIF-1α tends to decrease in hypoxic samples, while the down-regulation is significant when cells were exposed to 1 mM DMOG (Figure 7A). Prolyl hydroxylases and VHL gene expression are little affected by exposure to NO (Figure 7B,C).

**Figure 8:**
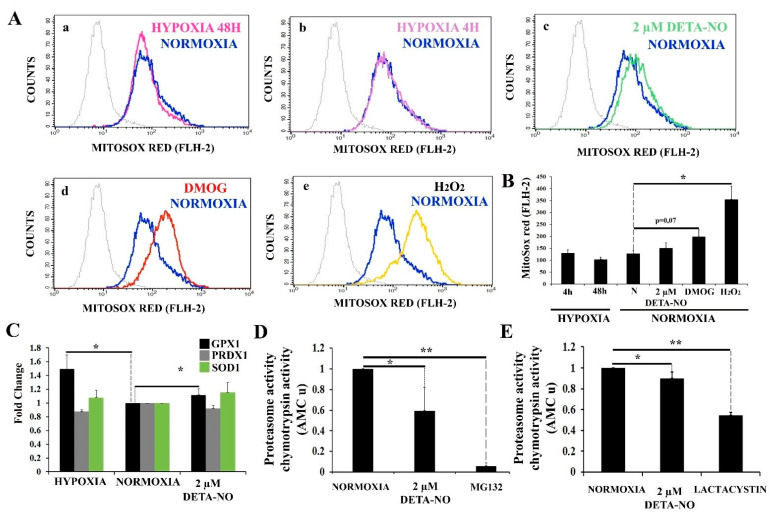
Treatment with low doses of nitric oxide donor maintains stable levels of mROS. (**A**) Measurement of O_2_^−^ levels with MitoSox red. Human iPSCs were cultured for 6 days and then exposed to either hypoxia or normoxia for 4 and 48 h. A different set of cells were exposed to 2 μM DETA-NO under normoxia for 48 h. Finally, another set of cells were exposed to DMOG (1 mM) for 4 h under normoxia. To induce a high O_2_^−^ production, cells cultured in normoxia were exposed to 200 μM H_2_O_2_ for 1 h. Cytometry plots represent cells counted versus red fluorescence emitted in the different treatments (a) hypoxia 48 h, (b) Hypoxia 4 h, (c) DETA-NO, (d) DMOG, and (e) H_2_O_2_ against the normoxia control. (**B**) The bar graph represents the mean intensity fluorescence ± SEM from 3 independent experiments. N, normoxia. (*) *p* < 0.005. (**C**) Analysis of the expression at the level of mRNA of antioxidant enzymes GPX1 (glutathione peroxidase 1), PRDX1 (peroxiredoxin 1), and SOD1 (superoxide dismutase 1) following exposure to hypoxia or 2 μM DETA-NO under normoxia for 48 h. The graph shows the mean of the relative expression of 5 independent experiments ± SEM. The relative expression was calculated with the algorithm ∆∆ Ct. Values were relativized to the β-actin mRNA expression level and normoxia sample. (*) *p* < 0.05. (**D**) Proteasome activity in MSUH-001 cells. 20S proteasome activity was measured by analyzing chymotrypsin-like activity in cell lysates. Cells were cultured for 6 days in a conditioned medium supplemented with bFGF and in the presence or absence of 2 μM DETA-NO for 48 h. MG132 (10 μM) was added to cell extracts to inhibit proteasome activity. Bars are means ± SEM of 4 independent experiments. (*) *p* < 0.05; (**) *p* < 0.005. (**E**) Proteasome activity in hESCs. 10 μM lactacystin was used as a proteasome inhibitor. Bars are means ± SEM of 4 independent experiments. (*) *p* < 0.05; (**) *p* < 0.005.

We thus sought to further explore the process responsible for NO-induced HIF-1α accumulation. It has been reported that mitochondrial ROS (mROS) enhances HIF-1α levels by inhibiting PHDs. mROS levels were thus studied with the fluorescent probe MitoSOX Red in human ESCs. The results show that 2 μM DETA-NO does not increase mROS production after treatment as compared to the normoxia control (Figure 8A panels a,b,c,d,e, and 8B). Exposure to DMOG led to a higher but non-significant increase in mROS production. Besides, hypoxia (4 h and 48 h) does not affect mROS production when compared to normoxia in hiPSCs. Here, cells were exposed to 200 μM H_2_O_2_ that served as a ROS source for a positive control. (Figure 8B). Next, we studied the effect of NO on the expression of antioxidant enzymes in hiPSCs. mRNA levels of glutathione peroxidase 1 (GPX1), peroxiredoxin 1 (PRDX1), and superoxide dismutase 1 (SOD1) were analyzed. A significant increase in GPX1 expression in cells cultured under hypoxia and in cells exposed to 2 μM DETA-NO for 48 h was observed when compared with the normoxia control (Figure 8C). On the other hand, mRNA PRDX1 and SOD1 were not altered by these experimental manipulations.

Furthermore, 20S proteasome activity under normoxia conditions and upon treatment with 2 μM DETA-NO was analyzed. Exposure of normoxic hiPSC to 2 μM DETA-NO led to a significant reduction in 20S proteasome activity (Figure 8D). Furthermore, 2 μM DETA-NO decreases proteasome activity of lysates obtained from hESC cultured in normoxic conditions (Figure 8E). As positive controls, proteasome inhibitors MG132 (MSUH-001 line) and lactacystin (HS181 line) were used.

## 4. Discussion

Hypoxic environments are considered appropriate for both maintenance of stemness and differentiation of PSCs [14,27]. We thus aimed to explore here the action of NO on the hypoxia response in human PSCs, since we had shown that low levels of NO contributes to the maintenance of stemness in mouse SCs by regulating pluripotency genes, energy metabolism, and mitochondrial function [26]. Several studies have reported the effect of NO on HIF-1α metabolism [28,29,30,31,32,33]. Moncada et al. found that induction of HIF-1α by NO could be both dependent on and independent of actions at the mitochondria [34]. They also showed that NO-induced HIF-1α accumulation was not dependent on O_2_ tension. We document here that exposure to low NO leads also to HIF-α protein accumulation in normoxic PSCs. Specifically, we determined that treatment with DETA-NO 2 μM for 48 h in normoxia induces stable levels of HIF-1α and HIF-2α, which were similar to those found in cells cultured under hypoxia. We thus proceeded to characterize the hypoxia-like effect of low NO in the maintenance of pluripotency and the regulation of mitochondrial and metabolic function in human PSCs. The dependence of PSCs on anaerobic metabolism for ATP production could then be a physiological adaptation to the low concentrations of O_2_ in vivo, since hypoxia is a common feature of the stem cell niche and the basis for the maintenance of the undifferentiated state [35]. Furthermore, it has been established that HIF-2α activates the expression of OCT-4, a master gene of pluripotency [36], and that HIF-1α and HIF-2α are required in the early stages of reprogramming of somatic cells, since they direct the necessary metabolic changes for the recovery of the pluripotent state [19,20]. We show here a significant increase in the expression of NANOG and OCT-4 mRNA and protein levels in cells treated with 2 μM DETA-NO when compared with normoxia conditions. Moreover, an increase in BrdU incorporation in cells treated with low NO was apparent, thus suggesting that a hypoxia-like state generated by low NO favors proliferation and pluripotency of PSCs under low oxygen tensions.

Several studies have documented that PSCs exhibit a high dependence on glycolysis under aerobic conditions, a feature shared with cancer cells [2,3,4,8,37]. During differentiation, a shift towards aerobic metabolism takes place, but the mechanisms involved in this transition have not been fully established. Additionally, it has been reported that overexpression of HIF-1α and HIF-2α allows the metabolic change necessary for cellular reprogramming, with enhanced expression of glycolytic genes [5,19,20]. We thus found it appropriate to evaluate the effect of NO on metabolic regulation in PSCs. The results show that expression of HIF target genes HK2, LDHA, PDK1, and PKM2 and angiogenesis-promoting gene VEGF-A is upregulated in hPSCs exposed to DETA-NO under normoxic conditions when compared to cells cultured under normoxia alone. Similar findings were observed in cells grown in hypoxia. These results are in line with those reporting a relevant role for anaerobic glycolysis in the maintenance of cell stemness, a condition that is promoted by upregulation of glycolytic HK2 and PKM2 gene expression by OCT-4 in a self-sustained cycle [5,38,39]. We also studied the impact of NO on mitochondrial metabolism and dynamics. The results reported here on mitochondrial oxidative metabolism are compatible with the downregulation of OXPHOS and a shift towards mitochondrial fission events. Previously, it has been reported that low doses of NO induce a reversible inhibition of COX, reducing OCR and ATP production due to a block of electron flux at Complex IV in normal and transformed cells [40]. In our study, we show for the first time, to the best of our knowledge, that low doses of NO donor DETA-NO decrease OCR and increase ECAR in human PCs.

A role for the fission protein DRP1 in the maintenance of iPSCs has been highlighted by the finding that pharmacological inhibition of DRP1 alters the typical morphology of iPSCs and drastically decreases alkaline phosphatase staining [5,41]. The data reported here indicate that DRP1 expression increases after treatment with low doses of NO in normoxia. Additionally, we observed that MNF2 expression decreases after treatment with low doses of NO under normoxia, as occurs under hypoxia. Increased DRP1 expression and activation is associated with low mitochondrial functionality, a feature of undifferentiated cells; therefore, this finding supports the role of NO as an inducer of pluripotency in hPSCs [5]. The analysis of mitochondrial function was further implemented by recording membrane potential (Δψm). The results reported here show that Δψm significantly decreases upon exposure of cells to NO. We also found that exposure to NO does not increase mROS levels and increased the expression of the antioxidant enzyme GPX1. These findings favor the notion that NO strengthens SC response to oxidative stress and prevents spontaneous differentiation events [10,42]. Furthermore, exposure of PSCs to NO under normoxic conditions promotes a lower rate of oxygen consumption and a higher extracellular acidification rate than hypoxia conditions, thus suggesting that the nitrogen radical is instrumental in promoting the glycolytic profile of PSCs and in preventing HIF degradation during spurious sample re-oxygenation after hypoxia culture.

HIF-1α is mainly regulated by protein degradation in a process dependent on hydroxylation [43,44]. Our results show that exposure to NO significantly decreases P402 hydroxylation in normoxic cells. It has been reported that NO stabilizes HIF-1α by S-nitrosylation of thiol groups and also by direct inactivation of PHDs [45,46]. We also found reduced proteasomal activity in NO-exposed PSCs. In this regard, it has been reported that NO, through S-nitrosylation or O-Glc-N-acetylation, reduces proteasomal degradation [47,48]. All in all, our results indicate that NO promotion of stemness depends on HIF1-α stabilization and activation of the cellular response to hypoxia, regulating energy metabolism, mitochondrial functionality, and the maintenance of pluripotency in hPSCs.

Regarding the role of NO as a hypoxia inducer, controversy exists as to whether it promotes HIF-1α stabilization [29,45] or HIF- 1α destabilization. We report here that low NO promotes HIF-1α stabilization under normoxic conditions. On the other hand, we and others have reported that high NO promotes SC differentiation and apoptosis [49]. Thus, the evidence gathered so far favors the idea of a dual action of NO in SC biology, a pro-survival and pro-stemness action at low NO and a pro-differentiating action at higher concentrations. From a practical point of view, low NO could then be used as a surrogate for hypoxic conditions in large-scale growth of PSCs.

## 5. Conclusions

**Figure 9:**
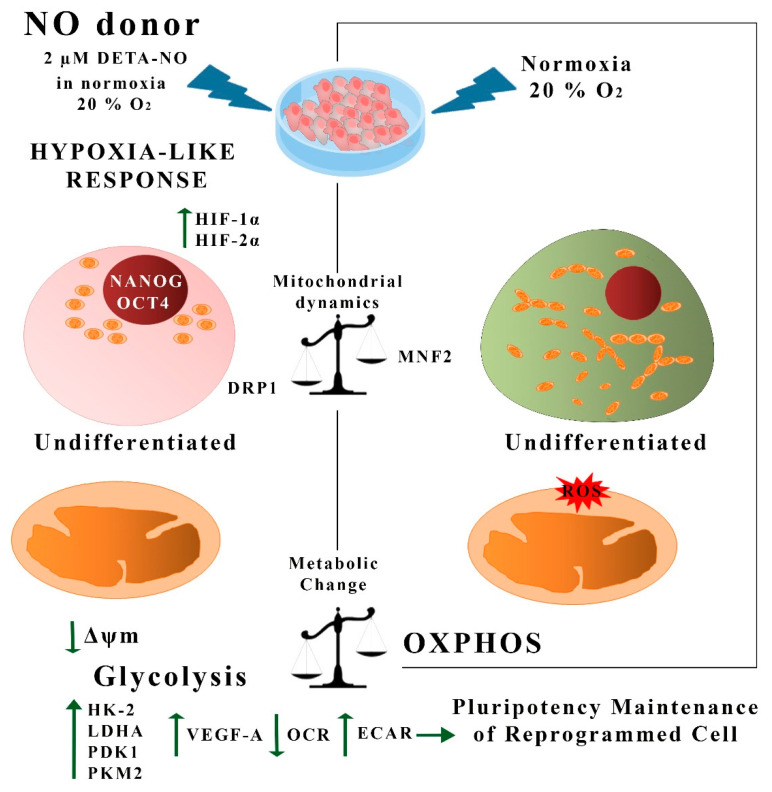
Summary of hypoxia-like actions of NO in human PS cells. In this study, we show that treatment with low doses of DETA-NO in normoxia induces a response similar to hypoxia in PSCs. Thus, exposure to 2 μM DETA-NO led to the accumulation of HIF-1α and HIF-2α proteins, enhanced expression of pluripotency genes NANOG and OCT-4, and a shift towards the expression of genes favoring anaerobic metabolism and tissue reoxygenation. Mitochondrial function and dynamics were also affected, with a decrease in O_2_ consumption and Δψm, an increase in DRP1 expression, and a decrease in MFN2. These actions are not dependent on changes in mROS levels. All in all, the results provided here indicate that NO induces a hypoxia-like response, regulating mitochondrial functionality and metabolic parameters in human PSCs, thus favoring pluripotency. NO (nitric oxide); ROS (reactive oxygen species); OCR (O_2_ consumption rate); ECAR (extracellular acidification rate), OXPHOS (oxidative phosphorylation).

The data presented in this report show that NO acts as an inducer of a hypoxia-like response in human PS cells, as summarized in Figure 9. It has been previously reported that the hypoxia condition helps to prevent spontaneous PSC differentiation in culture. The generation of low oxygen tension conditions might pose a challenge for the expansion of this type of SC at a high scale regarding cost and re-oxygenation events. We propose here that chemical NO donors at low doses might be instrumental when expanding PSCs under normoxia conditions. Additionally, studies carried out in our laboratory on the formulation of a xeno-free, chemically defined culture medium supplemented with DETA- NO show that stemness properties are greatly improved even at lower concentrations of growth factors, such as LIF or FGF. All in all, these findings provide a novel approach that deserves to be tested in the high-scale expansion of stem cells under GMP conditions.

## Data Availability

Data is contained within the article or supplementary material.

## Supplementary Materials

The following are available online at https://www.mdpi.com/article/10.3390/antiox10091408/s1: Figure S1: Low NO induces HIF-1α accumulation in human ESCs in normoxia. Figure S2: Low NO induces HIF-2α accumulation in human ESCs. Figure S3: Exposure to low doses of DETA-NO in normoxia increases the expression of pluripotency markers in human ESCs. Figure S4: 2 μM DETA-NO reduces red-JC-1 aggregation in human iPSCs. Table S1: List of primers.
