# Supplementary material for: Stemness of Human Pluripotent Cells: Hypoxia-Like Response Induced by Low Nitric Oxide
Source: Antioxidants (Basel). 2021 Sep 2;10(9):1408. doi: 10.3390/antiox10091408 (PMC8472328; doi:10.3390/antiox10091408)
Supplement: Supplementary file 1 [file antioxidants-10-01408-s001.zip › antioxidants-1347581-supplementary.pdf]

Supplementary Materials

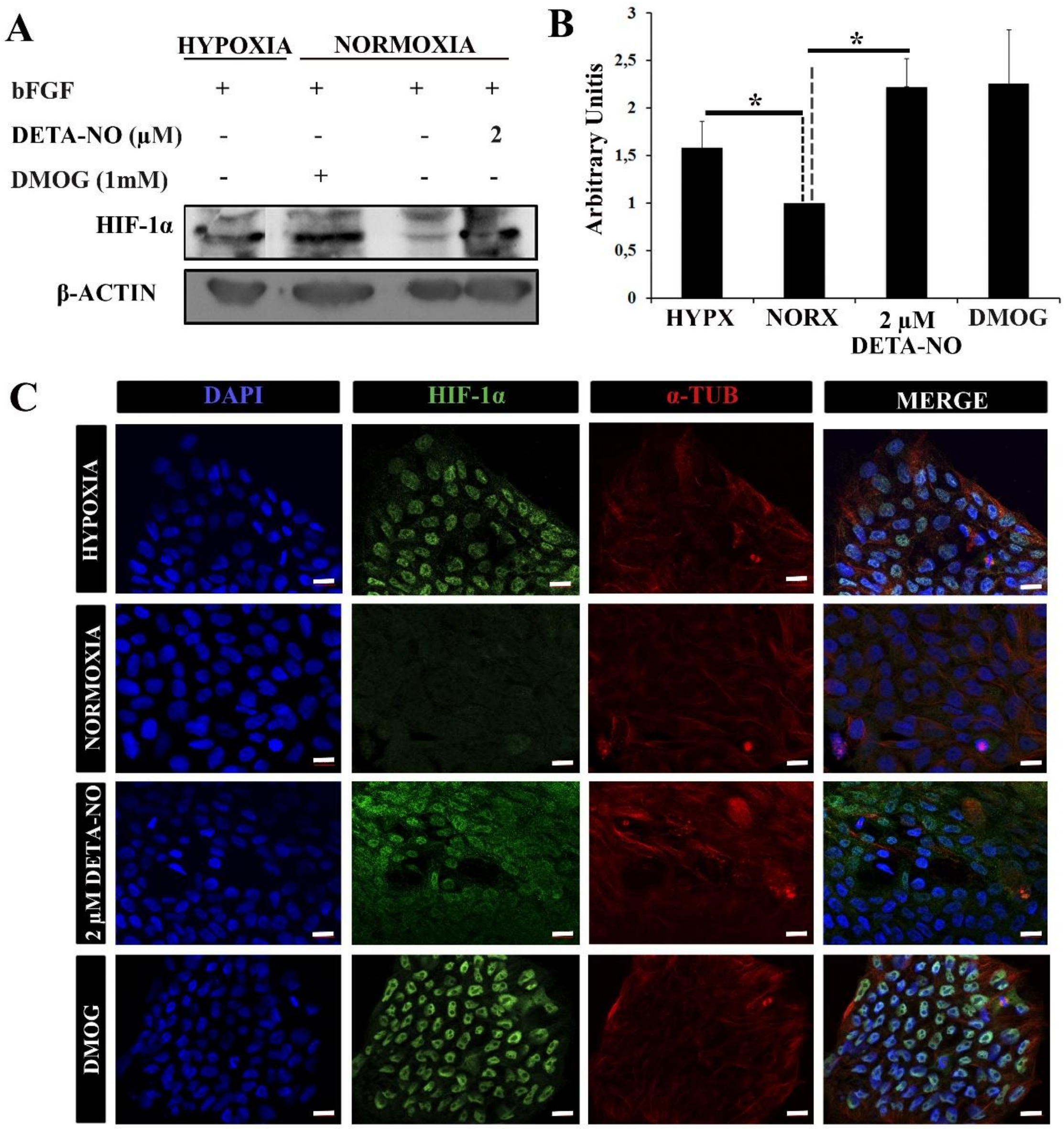

Figure S1. Low NO induces HIF-1α accumulation in human ESCs in normoxia. HS181 cells were cultured for 6 days in the presence of bFGF and exposed for 48 h to hypoxia (5% O_2), to 2 μM DETA-NO under normoxia for 48 h and 1mM DMOG for 4 h under normoxia. A) Analysis of the HIF-1α protein expression by Western blotting. As load control β-ACTIN was used. The figure is representative of 3 independent experiments. B) The bar graph represents the relative expression of HIF-1α in the different experimental conditions. HYPX (HYPOXIA), NORX (NORMOXIA). The expression values were relativized to the values in the normoxia control. Data are mean ± SEM of 3 independent experiments. C) HS181 cells expressing HIF-1α after different treatments in culture. Cells were labeled with DAPI (blue), anti-HIF-1α (green), and anti-α-TUBULIN (red). Scale bar: 20 μm. The images shown have been acquired with the Zeiss ApoTome Fluorescence Microscope.

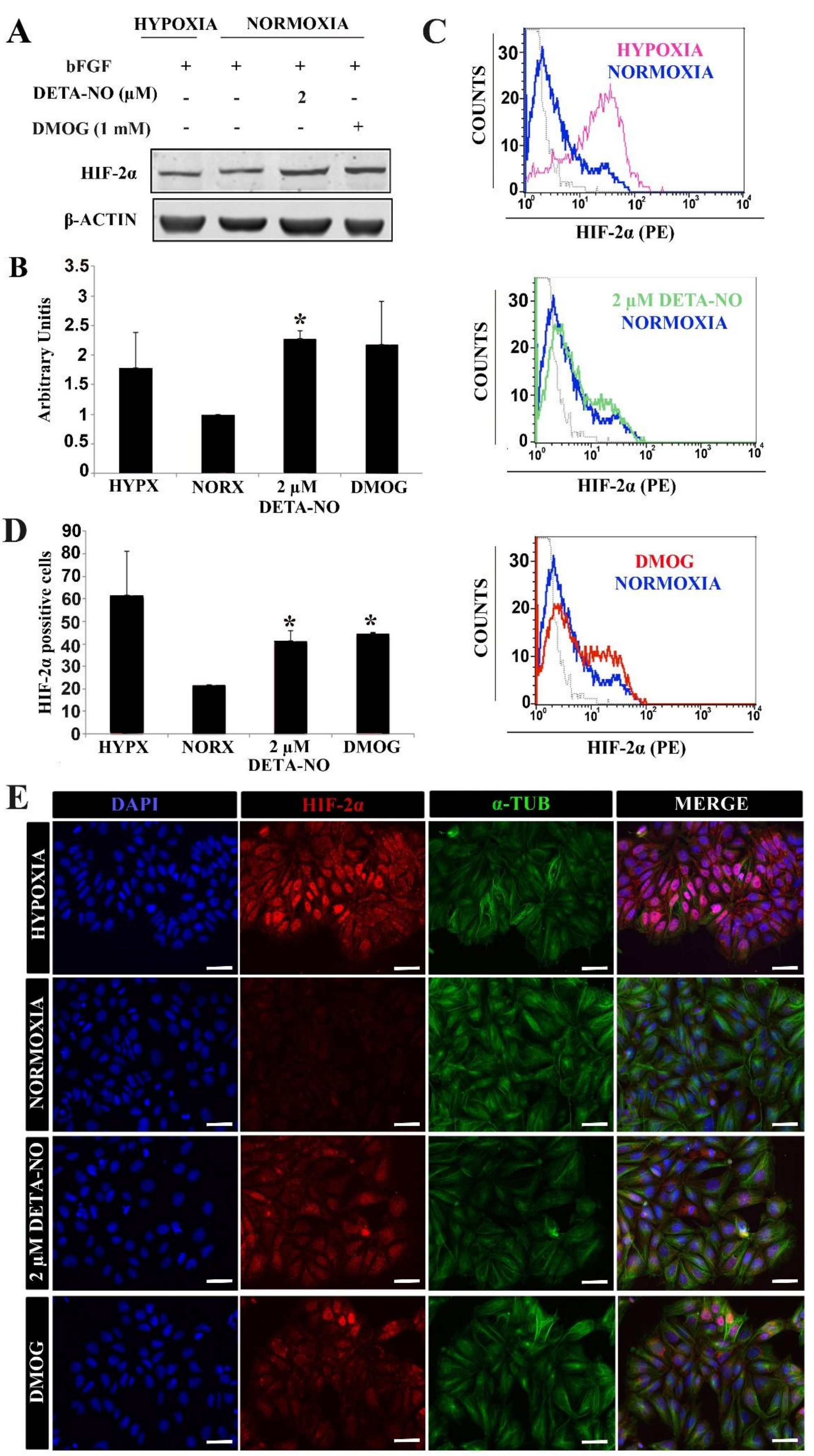

A
HYPOXIA
NORMOXIA
bFGF
DETA-NO (μM)
DMOG (1 mM)
HIF-2α
β-ACTIN
B
Arbitrary Unitis
HYPX
NORX
2 μM DETA-NO
DMOG
C
HYPOXIA
NORMOXIA
COUNTS
HIF-2α (PE)
2 μM DETA-NO
NORMOXIA
DMOG
NORMOXIA
D
HIF-2α possitive cells
HYPX
NORX
2 μM DETA-NO
DMOG
E
DAPI
HIF-2α
α-TUB
MERGE
HYPOXIA
NORMOXIA
2 μM DETA-NO
DMOG

Figure S2. Low NO induces HIF-2α accumulation in human ESCs. Exposure to low doses of DETA-NO induces accumulation of HIF-2α in normoxia. Human ESCs were cultured for 6 days in the presence of bFGF and then exposed hypoxia for 48 h, to 2 µM DETA-NO under normoxia and 4 h DMOG for 4 h1 mM under normoxia. A) Analysis of the expression of HIF-2α by Western blotting. β-ACTIN protein was used as load control. The figure is representative of 2 independent experiments. B) The bar graph represents the relative expression of HIF-2α in the different experimental conditions. Expression values were relativized to the condition of normoxia. The plot represents the mean ± SEM of 3 independent experiments. C) Quantification by flow cytometry. Fluorescence intensity corresponding to HIF-2α protein was plotted against the number of cells. Each plot is representative of 3 independent experiments. We used as negative controls the isotype control and secondary antibody control that is shown in the plot with gray lines. The number of cells analyzed is at least 5000 cells in each experimental condition. D) The percentage of HIF-2α positive cells ± SEM from 3 independent experiments is shown in the bar graph. E) Exposure to low doses of DETA-NO induces nuclear accumulation of HIF-2α in normoxia. In the figure, it can be observed cells expressing HIF-2α after different treatments in culture. Cells were labeled with DAPI (blue), anti-HIF-2α (red), and anti-α-TUBULIN (green). Scale bar: 50 µm. The images shown are representative of more than 10 planes per experimental condition and have been acquired with the Microscope Fluorescence Axio Imager Z2 microscope. (*) $p < 0.05$. HYPX (Hypoxia); NORX (Normoxia).

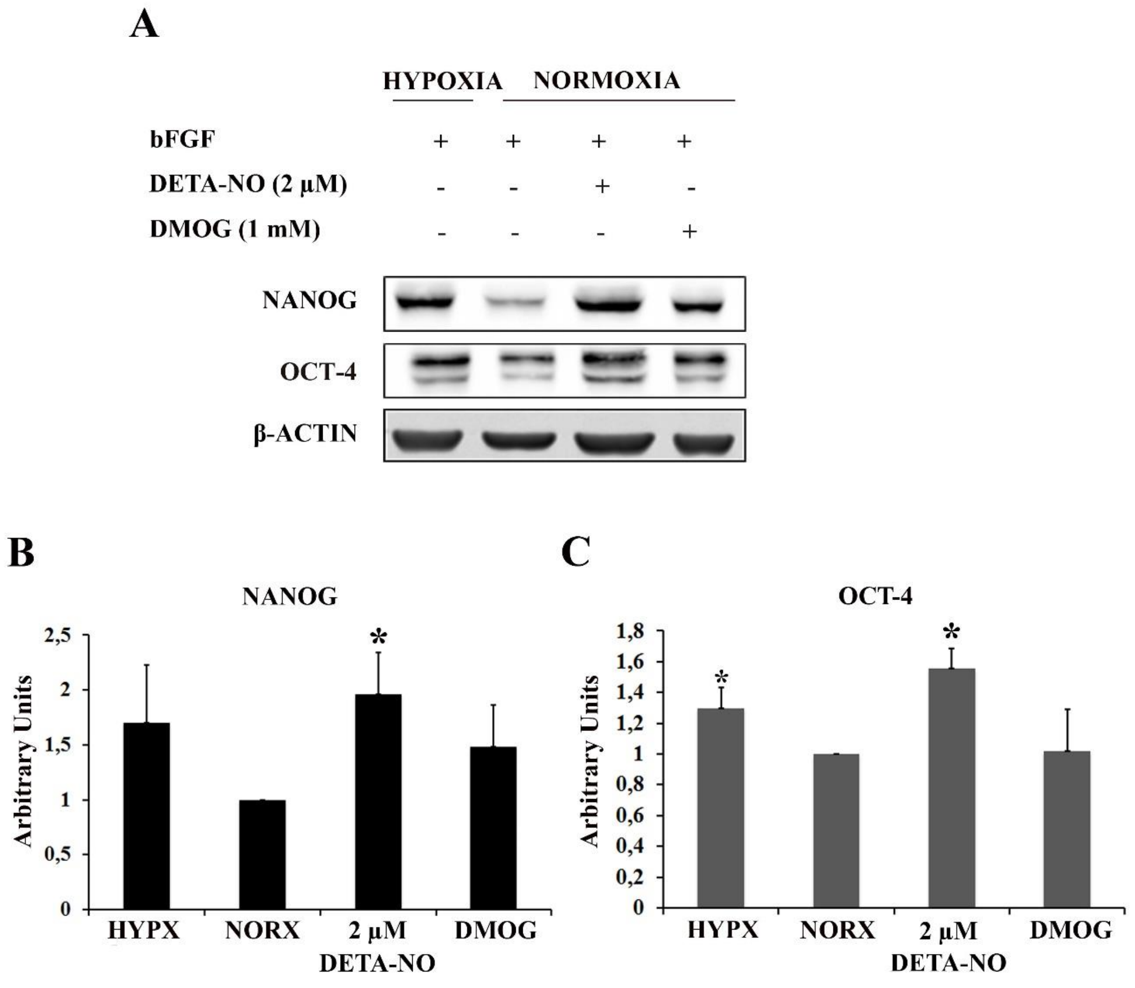

Figure S3. Exposure to low doses of DETA-NO in normoxia increases the expression of pluripotency markers in human ESCs. HS181 cells were cultured for 6 days in the presence of bFGF and then exposed to hypoxia for 48 h, to 2 μM DETA-NO under normoxia for 48 h or to DMOG 1 mM under normoxia for 4h. A) Analysis of the expression of NANOG and OCT-4 by Western blotting. As load control we used β-ACTIN. The figure is representative of 3 independent experiments. B) The bar graph represents the relative expression of NANOG in the different experimental conditions. The expression values were relativized to the normoxia condition. The plot represents the mean ± SEM of 3 independent experiments. C) The bar graph represents the relative expression of OCT-4 in the different experimental conditions. Expression values relativized to the condition of normoxia. The plot represents the mean ± SEM of 3 independent experiments. (*) p <0.05. HYPX (Hypoxia); NORX (Normoxia).

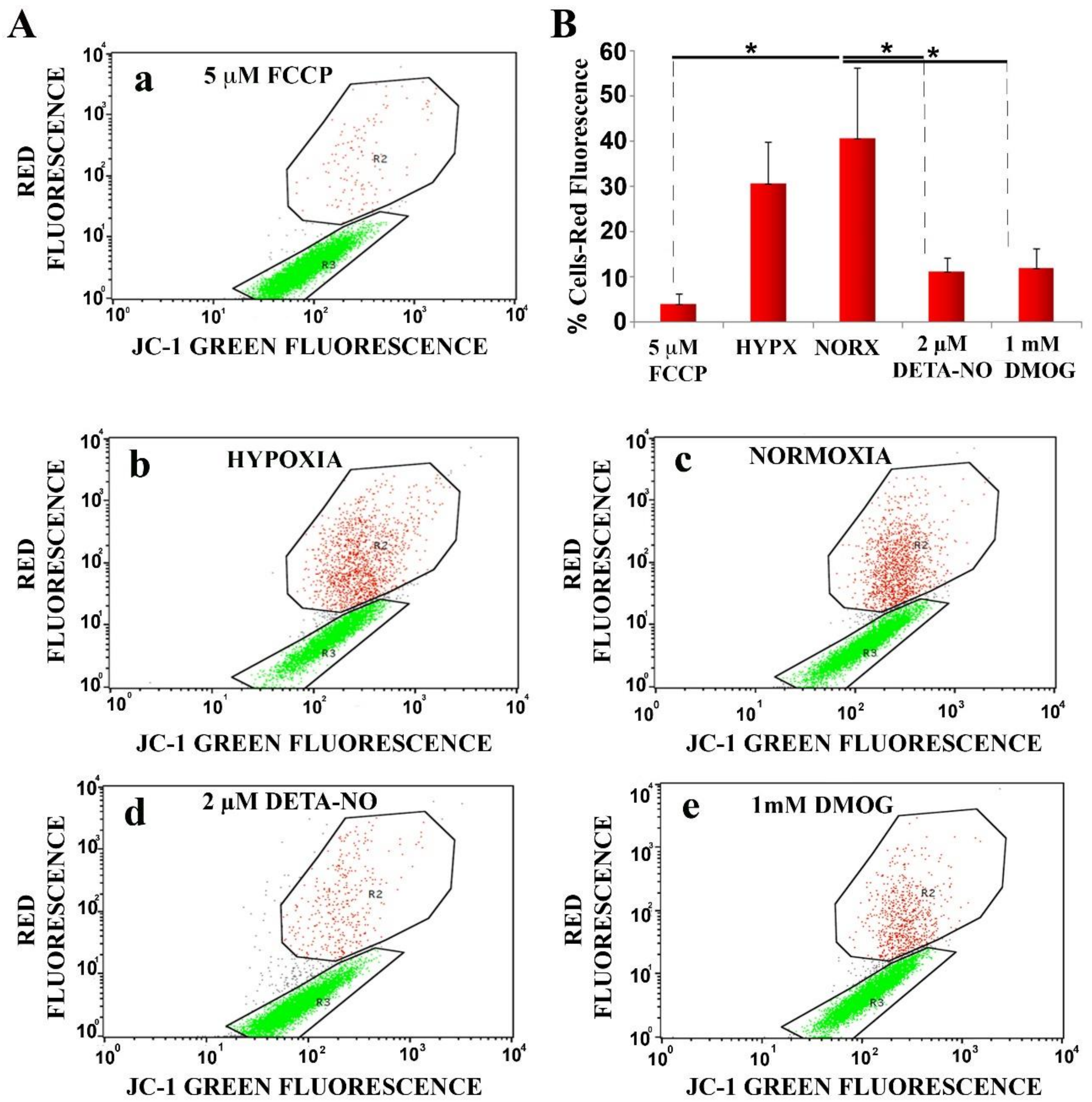

Figure S4. 2 μM DETA-NO reduces red-JC-1 aggregation in human iPSCs. Treatment with 2 μM DETA-NO decreases the percentage of cells with active mitochondria in normoxia.

Fluorescent staining of mitochondria with JC-1 in cells of the MSUH-001 line. Detection of Δψm by flow cytometry. A) Dot plots of cytometry in which it can be observed the red fluorescence emitted when JC-1 is aggregated against the green fluorescence emitted by the JC-1 monomers within the same cell population. In the a-e sections, the cytometry plots of the experimental conditions tested are shown. The population that emits red fluorescence is labeled as R2. B) The bar graph represents the percentage of fluorescence positive cells indicative of active hyperpolarized mitochondria with high Δψm. Data are mean ± SEM of 3 independent experiments. (*) $p < 0.05$. HYPX (HYPOXIA); NORX (NORMOXIA).

Supplementary Table S1. List of primers.

Gene	Sequences 5´- 3´
DRP1 F	GTGAACCCGTGGATGATAAA
DRP1 R	GAAACCTCAGGCACAAATAAAG
FIS1 F	AGCGGGATTACGTCTTCTA
FIS1 R	CCACGAGTCCATCTTTCTTC
GPX1 F	CCCTGCGGGGCAAGGTACTA
GPX1 R	GGGCATCAGGAGAACGCCAA
HK2 F	TAGCCTTCTTTGTGCGCCGT
HK2 R	GGTCAACCTTCTGCACTTGGTCA
LDHA F	TATGGAGTGGAATGAATGTTG
LDHA R	CCCTTAATCATGGTGGAAACT
MNF1 F	CTGGCTAAGAAGGCGATTAC
MNF1 R	TCCCACTAGGGAGAACTTTAT
MNF2 F	CTGAGACAGGACAGAAGAGA
MNF2 R	GATAGAGTTGCATCGAGAGAAG
NANOG F	CCCAAAGGCAACAACCCACT
NANOG R	GCTGGGTGGAAGAGAACACA
NRF1 F	ACTCGTGTGGGACAGCAAGC
NRF1 R	GAAGCTGGGCCTGGGTCATT
OCT4 F	TATGCAAAGCAGAAACCCTCG
OCT4 R	TTCGGGCACTGCAGGAACAAA
PDK1 F	TGC CTCTGGCTGGTTTTGGTTAT
PDK1 R	TGTCTAGGCACTGCGGAACG
PDKM2 F	ACTCACTCTGGGCTGTAA

PDKM2 R	CCTCCTTCTTCCCTTGATTG
PGC-1α F	TCAGTAAGGGGCTGGTTGCC
PGC-1α R	CAGCACACTCGATGTCACTCCA
PHD2 F	AGGCAAAGCCCAGTTTGCTGA
PHD2 R	ACCCTCACACCTTTTTCACCTGT
PHD3 F	GAGCCGGCTGGGCAAATACT
PHD3 R	GGATCCCACCATGTAGCTTGGC
PRDX1 F	GGACTGGGACCCATGAACATTCCTTT
PRDX1 R	TTGTCAGTGAACTGGAAGGCCTGAAC
SOD1 F	AGGCATGTTGGAGACTTGGGC
SOD1 R	TTTTTTCATGGACCACCAGTGTGCG
TFAM F	TGCGCTCCCCCTTCAGTTTT
TFAM R	TACCTGCCACTCCGCCCTAT
VEGF-A F	ACACATTGT TGGAAG AAGCAGCCC
VEGF-A R	AGGAAGGTCAACCACTCACACACA
VHL F	GCTACCGAGGTCACCTTTGGCTCT
VHL R	CGGACAACCTGGAGGCATCGC
β-ACTIN F	CGTACCACTGGCATCGTGAT
β-ACTIN R	TTCTCCTTAATGTCACGCAC
